# The Role of the Insulin/Glucose Ratio in the Regulation of Pathogen Biofilm Formation

**DOI:** 10.3390/biology12111432

**Published:** 2023-11-15

**Authors:** Balbina J. Plotkin, Scott Halkyard, Emily Spoolstra, Amanda Micklo, Amber Kaminski, Ira M. Sigar, Monika I. Konaklieva

**Affiliations:** 1 Department of Microbiology and Immunology, Midwestern University, Downers Grove, IL 60515, USA; sdhalkyard@gmail.com (S.H.); espoolstra93@midwestern.edu (E.S.); amanda.micklo@midwestern.edu (A.M.); akamin@midwestern.edu (A.K.); isigar@midwestern.edu (I.M.S.); 2 Department of Chemistry, American University, Washington, DC 20016, USA; mkonak@american.edu

**Keywords:** biofilm, multi-drug resistance, insulin receptor binding, hyperglycemia, trauma

## Abstract

**Simple Summary:**

Insulin and glucose affect the biofilm formation of both Gram-positive and Gram-negative bacteria over the physiologic range in a manner that is dependent on the ratio of insulin to glucose. These findings provide insight into the mechanism underpinning empirical sepsis management in trauma care.

**Abstract:**

During the management of patients in acute trauma the resulting transient hyperglycemia is treated by administration of insulin. Since the effect of insulin, a quorum sensing compound, together with glucose affects biofilm formation in a concentration-specific manner, we hypothesize that the insulin/glucose ratio over the physiologic range modulates biofilm formation potentially influencing the establishment of infection through biofilm formation. Methods: A variety of Gram-positive and Gram-negative bacteria were grown in peptone (1%) yeast nitrogen base broth overnight in 96-well plates with various concentrations of glucose and insulin. Biofilm formation was determined by the crystal violet staining procedure. Expression of insulin binding was determined by fluorescent microscopy (FITC-insulin). Controls were buffer alone, insulin alone, and glucose alone. Results: Overall, maximal biofilm levels were measured at 220 mg/dL of glucose, regardless of insulin concentration (10, 100, 200 µU/mL) of the organism tested. In general, insulin with glucose over the range of 160–180 mg/dL exhibited a pattern of biofilm suppression. However, either above or below this range, the presence of insulin in combination with glucose significantly modulated (increase or decrease) biofilm formation in a microbe-specific pattern. This modulation appears for some organisms to be reflective of the glucose-regulated intrinsic expression of bacterial insulin receptor expression. Conclusion: Insulin at physiologic levels (normal and hyperinsulinemic) in combination with glucose can affect biofilm formation in a concentration-specific and microbe-specific manner. These findings may provide insight into the importance of co-regulation of the insulin/glucose ratio in patient management.

## 1. Introduction

Hyperglycemia is a common clinical presentation that occurs in a significant number of critically ill individuals. For critically ill inpatients, hyperglycemia requires treatment when the blood glucose level is persistently >180 mg/dL. In trauma, transient hyperglycemia is a common physiologic response [1,2,3,4]. Standard ICU and surgical protocol are empirical administration of insulin to modulate the glucose levels, decreasing them to levels associated with reduced morbidity and mortality, i.e., below 220 mg/dL [5,6]. Other studies suggest tight glycemic endpoint control (via infusion) with the resultant blood glucose endpoint of between 150 mg/dL and 180 mg/dL [7]. This glycemic level appears to further improve patient outcomes in both pre- and post-operative settings [8]. Although this empirical treatment application has been shown to be effective, the underlying mechanism(s) associated with microbial sepsis under various glycemic conditions vis-à-vis insulin levels has not been determined. The state of sepsis, i.e., bacteremia, provides an opportunity for bacteria to colonize host surfaces and form biofilms. This formation of biofilms is an important characteristic of bacteria in sepsis since it acts as a protective mechanism against the hostile host environment, i.e., immune defense, as well as provides protection against any antimicrobial therapy that may be used in patient management [9]. Furthermore, these biofilms can play a key role as a locus for secondary spread with potentiation of systemic infection. Biofilm formation by microbes is highly adaptable to the environment and in general regulated through an organism’s response to quorum chemical signaling compounds, which include mammalian hormones, e.g., insulin.

Insulin is an interkingdom quorum chemical signaling molecule that modulates *Escherichia coli* (*E. coli*) adherence and biofilm formation [10,11,12]. Taxonomically production of insulin spans the Eubacteria (*E. coli*), Fungi (*Neurospora crassa*), Protist (*Tetrahymena*), Animalia (mammals), and, more controversially, Plantae (*Momordica charantia)* [13,14,15]. With respect to procaryotes, LeRoith, et al. [16] demonstrated that wild-type *E. coli* elaborates a native insulin with properties similar to mammalian insulin with respect to structure and function in a mammalian liver cell assay system. One function that insulin exhibits in *E. coli* is that of a rheostat, with respect to the expression of microbial phenotype regarding biofilm-forming behavior, i.e., adherence and chemotaxis, both of which appear to be dependent on the presence of carbohydrates, particularly glucose [10,17,18]. Although the administration of insulin is a mainstay in the management of the glycemic state in trauma and hospitalized patients, to date, the effect of insulin/glucose ratio over the general physiologic range on biofilm formation has not been determined. The focus of this study was to screen various Gram-positive and Gram-negative clinical isolates for biofilm formation in response to insulin/glucose levels over a physiological range to determine if there is a relationship between the clinically reported hyperglycemic–sepsis association and the resultant microbial response of biofilm formation.

## 2. Materials and Methods

Both standard ATCC highly stable quality control isolates and clinical isolates obtained from blood cultures were used for the study. All isolates were able to form biofilm under permissible environmental conditions. The bacterial isolates used were *E. coli* extended-spectrum beta-lactamase (ESBL) L108; *E. coli* ESBL L109; *Acinetobacter baumannii* L185; *A. baumannii* L186; *A. baumannii* L187; *Klebsiella pneumoniae* ATCC 27736; *K. pneumoniae* carbapenemase (KPC)-producing bacteria L133; *K. pneumoniae* ESBL L174; multi-drug-resistant (MDR) *K. pneumoniae* B249 (KLE B249); vancomycin-resistant *Enterococcus faecalis (E.faecalis)* 9 (VRE); methicillin-resistant *Staphylococcus aureus* (MRSA) isolates (X52960, M23110, M23304, W52559, T51995); and methicillin-sensitive *S. aureus* (MSSA) isolates (ATCC 25923, T55358, M24839). All non-ATCC isolates were reported as blood culture isolates from patients admitted to the hospital with various presenting acute ailments, e.g., trauma and acute infectious presentation, which necessitated a blood draw for bacterial culture and were a generous gift from the late Paul Schreckenberger.

Biofilm assay: Biofilm formation was measured using the standard quantitative crystal violet bacterial pellicle absorbance assay [19]. Organisms were maintained at −80 °C until use. After overnight growth on LB agar, organisms were inoculated to the same density (10^3^ CFU/mL) in LB broth with and without various concentrations of insulin (10, 100, 200 µU/mL) and/or glucose, and placed in flat bottom untreated tissue culture plates (150 µL/well) [10,13]. After overnight growth (18 h, 37 °C), the wells were carefully washed (4X: PBS; pH 7.0), air dried, and then stained with crystal violet (Troy Biologics, Troy, MI, USA). After extensive washing, the plates were dried, and then the stain was solubilized with absolute ethanol (300 µL/well). The dissolved stain was transferred to another 96-well flat bottom plate (200 µL/well) and the level of biofilm present was quantified by immediately measuring the optical density in an (EIA reader (Dynatech Laboratories, Inc., Chantilly, VA, USA); 590 nm). Positive controls for biofilm formation consisted of organisms grown in LB alone, organisms grown in insulin in LB alone, and organisms grown in glucose in LB alone. Experiments were conducted in octuplicate and repeated thrice (*n* = 24). Data were evaluated by analysis of variance (ANOVA; GraphPad InStat 3.06 for Windows, GraphPad Software Inc., Version 8.0.0, San Diego, CA, USA).

For *K. pneumoniae* ATCC 27736, a stable highly characterized encapsulated isolate used for antibiotic testing, capsule levels were also determined in duplicate plates stained with Alcian blue, a stain specific for acidic polysaccharides, (1% *w*/*v* PBS, 30 min, room temperature) [17,20,21]. The stain was dissolved in 300 µL/well pyridine (Sigma-Aldrich, St. Louis, MO, USA). The dissolved stain was transferred to another 96-well flat bottom plate (200 µL/well) and the level of the capsule was quantified by immediately measuring the optical density in an EIA reader at 450 nm.

Insulin binding: To determine if insulin intrinsically bound to each of the species screened for biofilm formation, a randomly chosen isolate was grown overnight (18 h, 37 °C) in LB alone and 10–15 μL of cell suspension were then removed, dried onto a glass slide, methanol fixed, and stained (30 min; 37 °C; moisture chamber) with fluorescein isothiocyanate (FITC)-labeled insulin (25 µg/mL PBS; Sigma-Aldrich). Slides were coded and examined by epifluorescence microscopy (Nikon E600 microscope). Cell fluorescence was determined from photomicrographs of representative fields (*n* = 3). FITC-insulin control consisted of cells pre-treated with insulin (Humulin R^®^; 1 U/mL) prior to staining with FITC-insulin (no insulin fluorescence observed for any of the isolates).

Statistical analysis: All assays were carried out in triplicate and repeated at least twice unless otherwise indicated. Whenever possible, experiments were coded and performed in a blind fashion. Analysis of variance was used to determine differences between experimental conditions with *p* < 0.05 considered significant. If statistical significance was found, a Tukey–Kramer post hoc analysis was applied (InStat3, GraphPad Software, San Diego, CA, USA).

## 3. Results

### 3.1. Insulin/Glucose Concentration Effect on Biofilm Formation by MSSA, MRSA, and VRE

**Figure 1:**
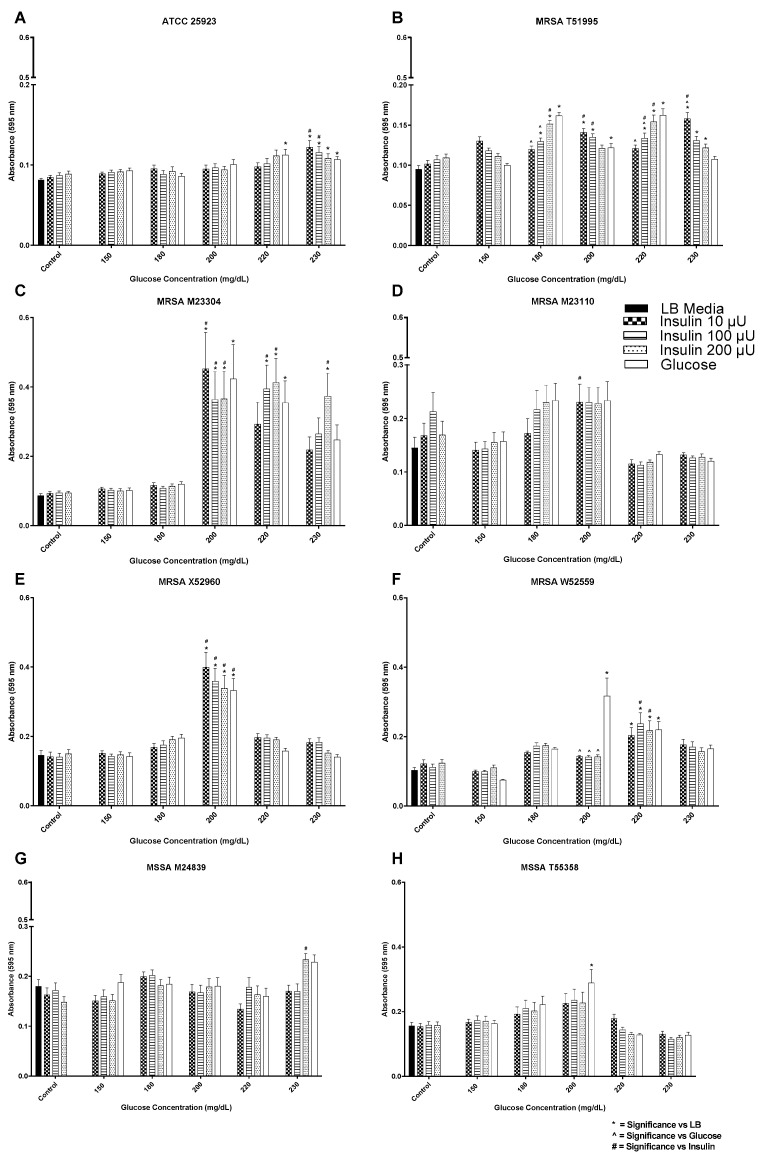
Biofilm formation by methicillin-sensitive (**A**–**C**) *S. aureus* (MSSA) isolates and various clinical MRSA isolates (**D**–**H**) in response to a range of insulin/glucose concentrations. * Significantly different (*p* ≤ 0.05) as compared to LB. ^˄^ Significantly different (*p* ≤ 0.05) as compared to glucose. ^#^ Significantly different (*p* ≤ 0.05) as compared to insulin at homologous concentration. Positive controls for biofilm formation were growth in LB alone, each insulin concentration in LB alone, and glucose in LB alone.

**Figure 2:**
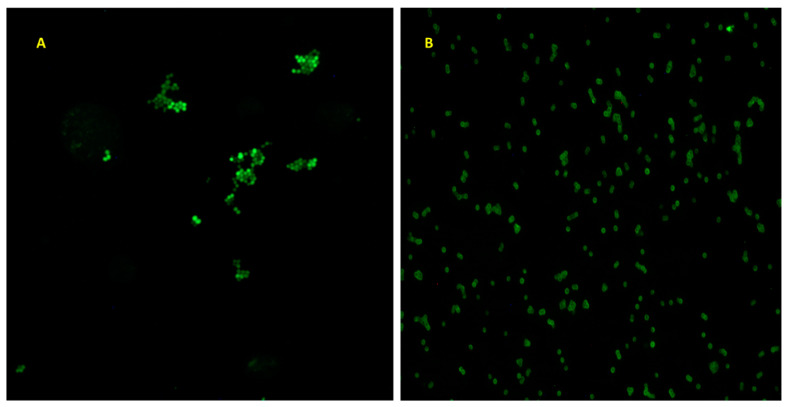
Binding of FITC-insulin to *S. aureus* ATCC 25923 MSSA (**A**) and *S. aureus* T51995 MRSA (**B**) cells after 18 h of aerobic growth.

Seven clinical isolates (5 MRSA and 2 MSSA) and one standard control MSSA strain were screened for biofilm formation in response to insulin and glucose (Figure 1A–H). Regardless of the isolation source, i.e., blood, the level of biofilm expressed by the various isolates was heterologous. For the stable MSSA isolate ATCC 25923 (Figure 1A), biofilm levels maximized at or above the insulin/glucose ratio of 200 µU/mL insulin and 220 mg/dL glucose. MSSA clinical isolates T55358 and M24839 (Figure 1B,C) biofilm levels were not significantly affected by the presence of insulin, regardless of concentration tested with biofilm level being a general reflection of glucose level. The exception to this trend was that of insulin/glucose ratio of 200 µU/mL insulin for T55358, where biofilm formation was enhanced, and 230 mg/dL glucose for M24839, where its biofilm level was suppressed when either 10 or 100 µU/mL insulin was present, as compared to 230 mg/dL glucose alone. For isolates X52960, M23110, and M23304, insulin had no significant effect on biofilm formation as compared to that of glucose, although maximal biofilm levels were measured at or around 200 mg/dL glucose (Figure 1D–F). Of interest is the glucose concentration specificity for MRSA X52960, which exhibited its maximal peak biofilm level at 200 before decreasing (at 230 mg/dL) to levels similar to those measured for 150 and 180 mg/dL glucose. In contrast, insulin at all concentrations suppressed MRSA W52559 maximal glucose biofilm levels (200 mg/dL glucose) while conversely enhancing biofilm production as compared to glucose alone (150 mg/dL) (Figure 1G). The greatest insulin concentration effect per glucose concentration was measured for MRSA T51995. These findings indicate that biofilm formation in response to insulin/glucose ratios was isolate, insulin, and glucose concentration dependent, although overall, the maximal biofilm level was measured at or around 200 mg/dL, regardless of insulin level at that glucose concentration. In addition to the ability to respond to insulin, randomly chosen isolates (Figure 2) also exhibited the ability to intrinsically bind FITC-insulin. Interestingly, the overall pattern of binding for the laboratory strain MSSA ATCC 25923 displayed cocci in clusters with variable levels of fluorescent intensity. In contrast, MRSA T51995 fluorescence was localized to the coccal margins for a majority of the cells.

**Figure 3:**
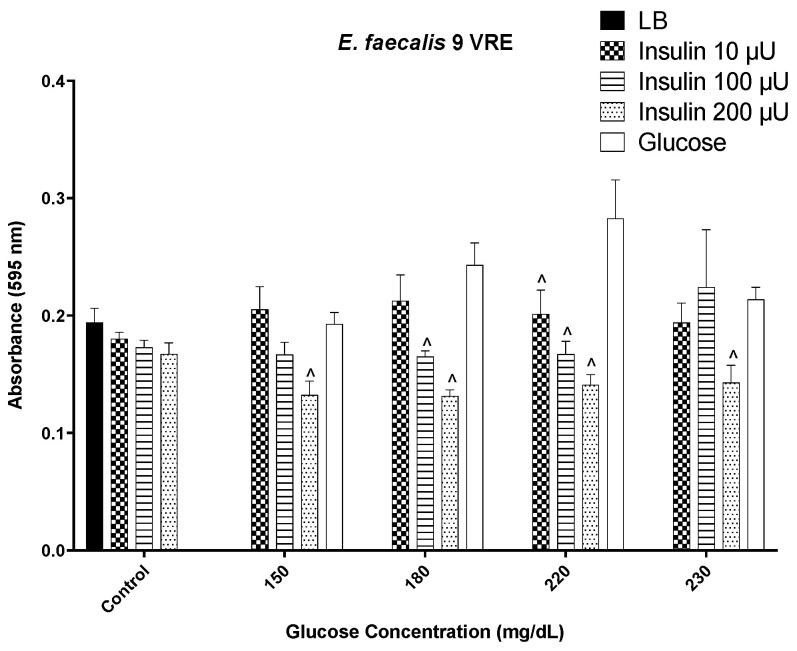
Biofilm formation by a vancomycin-resistant *E. faecalis* 9(VRE) isolate in response to a range of insulin/glucose concentrations. ^˄^ Significantly different (*p* ≤ 0.05) as compared to glucose. Positive controls for biofilm formation were growth in LB alone, each insulin concentration in LB alone, and glucose in LB alone.

**Figure 4:**
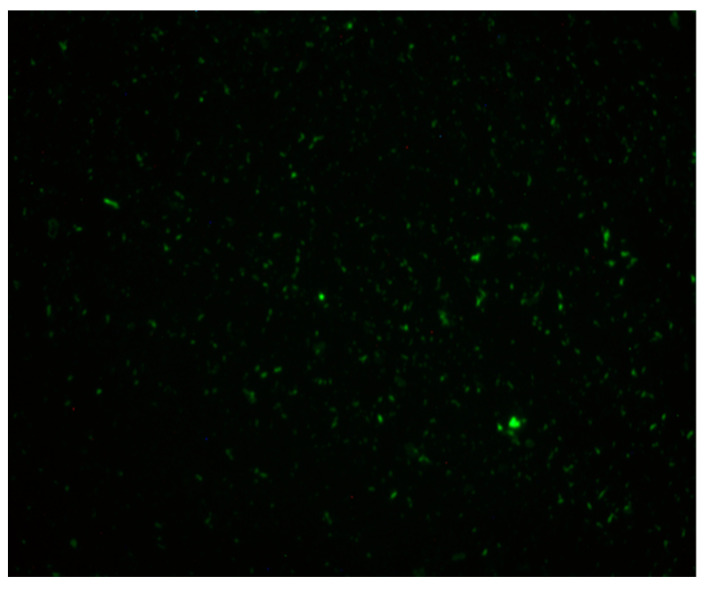
Binding of FITC-insulin to *E. faecalis* 9 VRE cells after 18 h of aerobic growth.

Vancomycin-resistant *E. faecalis* exhibited a pattern of biofilm formation that was insulin concentration sensitive, with the greater the insulin level, the more controlled the level of biofilm. Although biofilm levels increased with increasing glucose alone, the presence of insulin maintained biofilm levels at a constant level through 220 mg/dL glucose (Figure 3). However, at 230 mg/dL glucose, insulin’s ability to suppress biofilm formation was similar to glucose at 100 µU/mL insulin. This isolate exhibits intrinsic ability to bind FITC-insulin in a varied pattern (Figure 4).

### 3.2. Insulin/Glucose Concentration Effect on Biofilm Formation by Gram-Negative Pathogens

**Figure 5:**
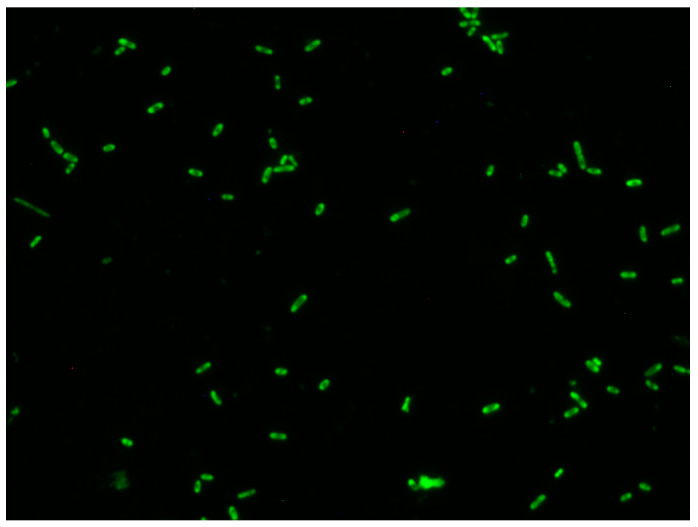
Binding of FITC-insulin to *E. coli* ESBL L108 cells after 18 h of aerobic growth in LB broth.

**Figure 6:**
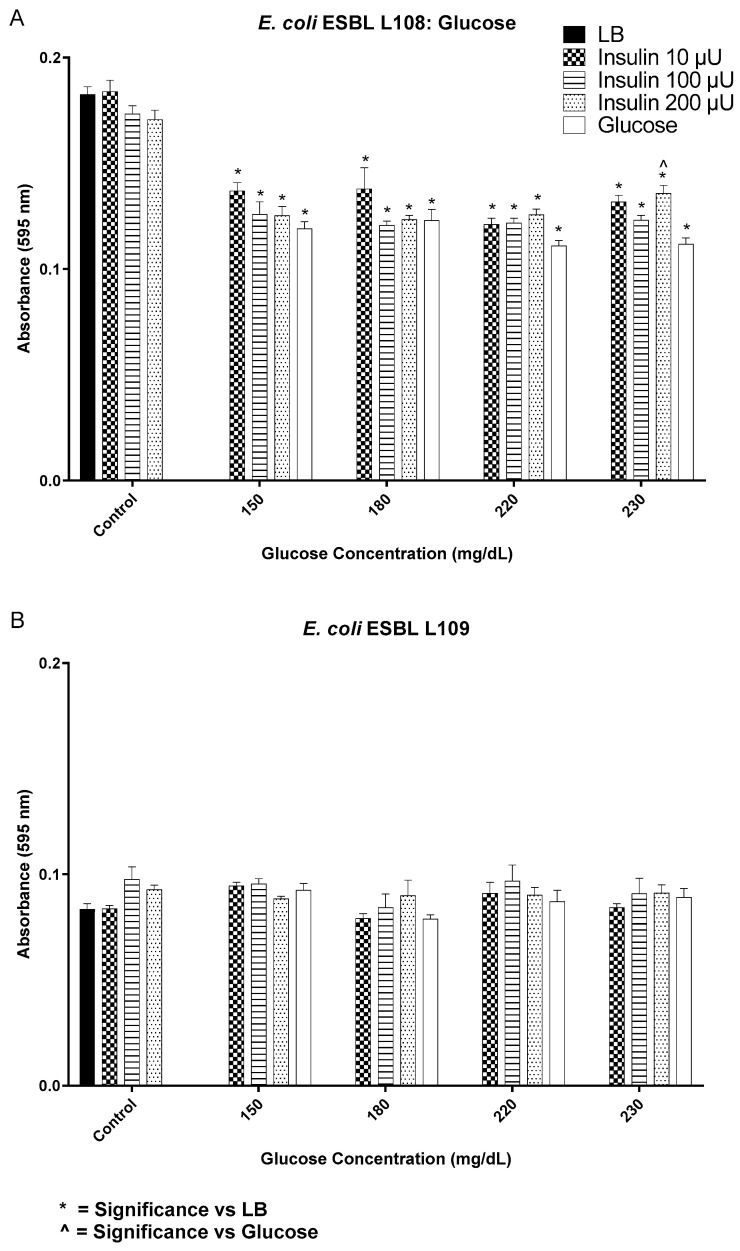
Biofilm formation by *E. coli* isolates in response to a range of insulin/glucose concentrations for two clinical multi-drug resistant isolates (**A**,**B**). * Significantly different (*p* ≤ 0.05) as compared to LB. ^˄^ Significantly different (*p* ≤ 0.05) as compared to glucose. Positive controls for biofilm formation were growth in LB alone, each insulin concentration in LB alone, and glucose in LB alone.

Intrinsic binding of FITC-insulin to *E. coli* L108 resulted in a varied pattern of fluorescence (Figure 5). Cells exhibited mono- and bi-polar capping, while others showed an even or punctate pattern of fluorescence. For *E. coli* ESBL 108 at 220 and 230 mg/dL glucose, all concentrations of insulin induced significantly more biofilm as compared to glucose alone, while at the lowest insulin concentration tested (10 µU/mL), there was enhanced biofilm production for 150 and 180 mg/dL glucose concentrations (Figure 6A). In contrast, for *E. coli* ESBL L109 insulin/glucose effect on biofilm expression, there was no significant difference in biofilm level for 10, 100, and 200 µU/mL insulin regardless of glucose concentration (Figure 6B).

**Figure 7:**
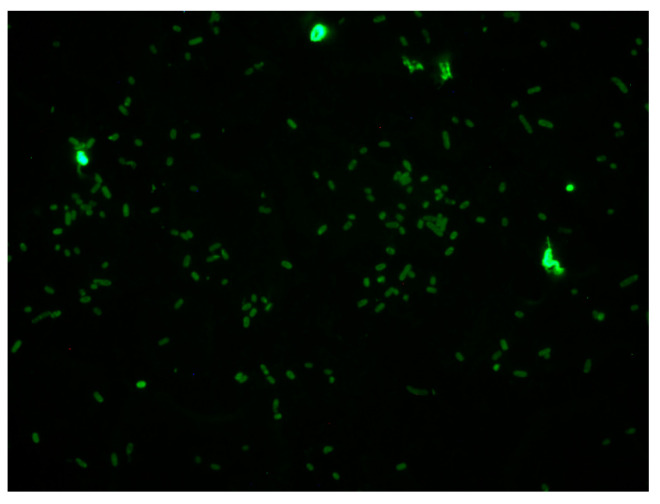
Binding of FITC-insulin to aerobically grown *A. baumannii* L187 cells after 18 h of aerobic growth.

**Figure 8:**
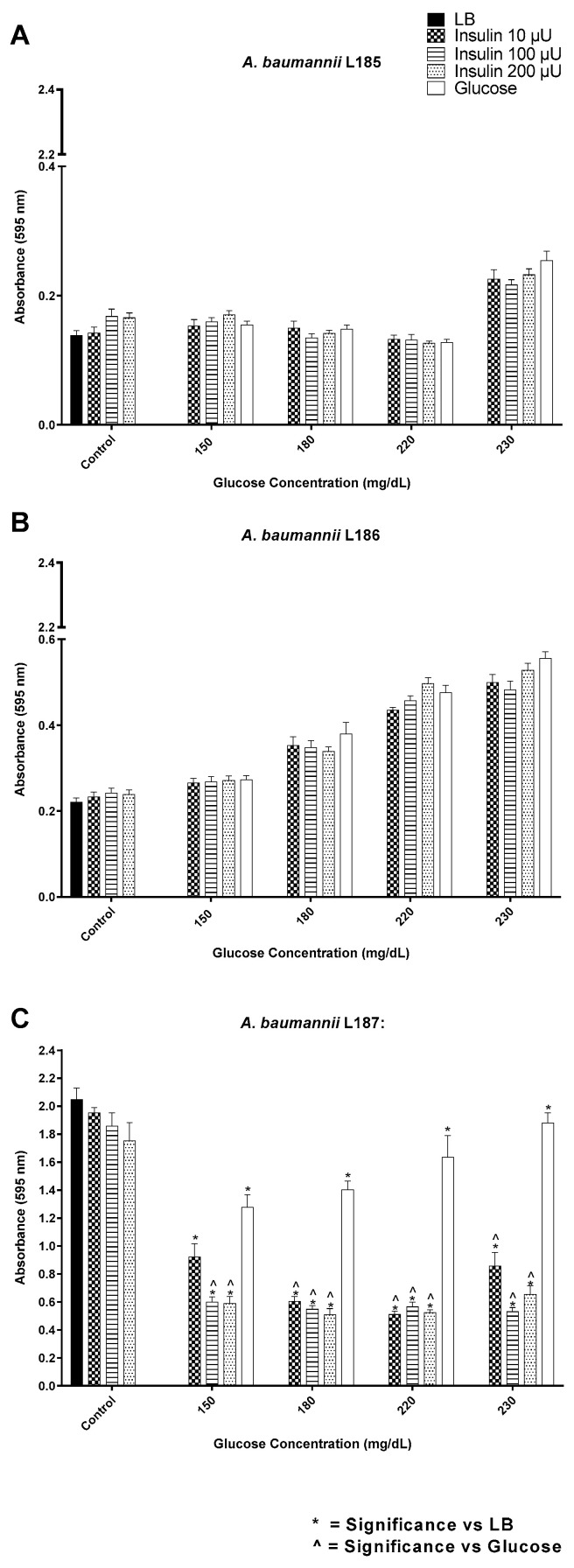
Biofilm formation by *A. baumannii* multi-drug resistant clinical isolates (**A**–**C**) in response to a range of insulin/glucose concentrations. * Significantly different (*p* ≤ 0.05) as compared to LB. ^˄^ Significantly different (*p* ≤ 0.05) as compared to glucose. Positive controls for biofilm formation were growth in LB alone, each insulin concentration in LB alone, and glucose in LB alone.

Similar to what was observed for *E. coli*, *A. baumannii* L187 exhibits intrinsic binding of insulin in various patterns, e.g., polar, punctate, and even (Figure 7). Insulin had no significant effect on biofilm formation for isolates L185 and L186 (Figure 8A,B). In contrast, for isolate L187, insulin at all concentrations tested significantly suppressed biofilm formation as compared to glucose alone control for all glucose concentrations (Figure 8C).

**Figure 9:**
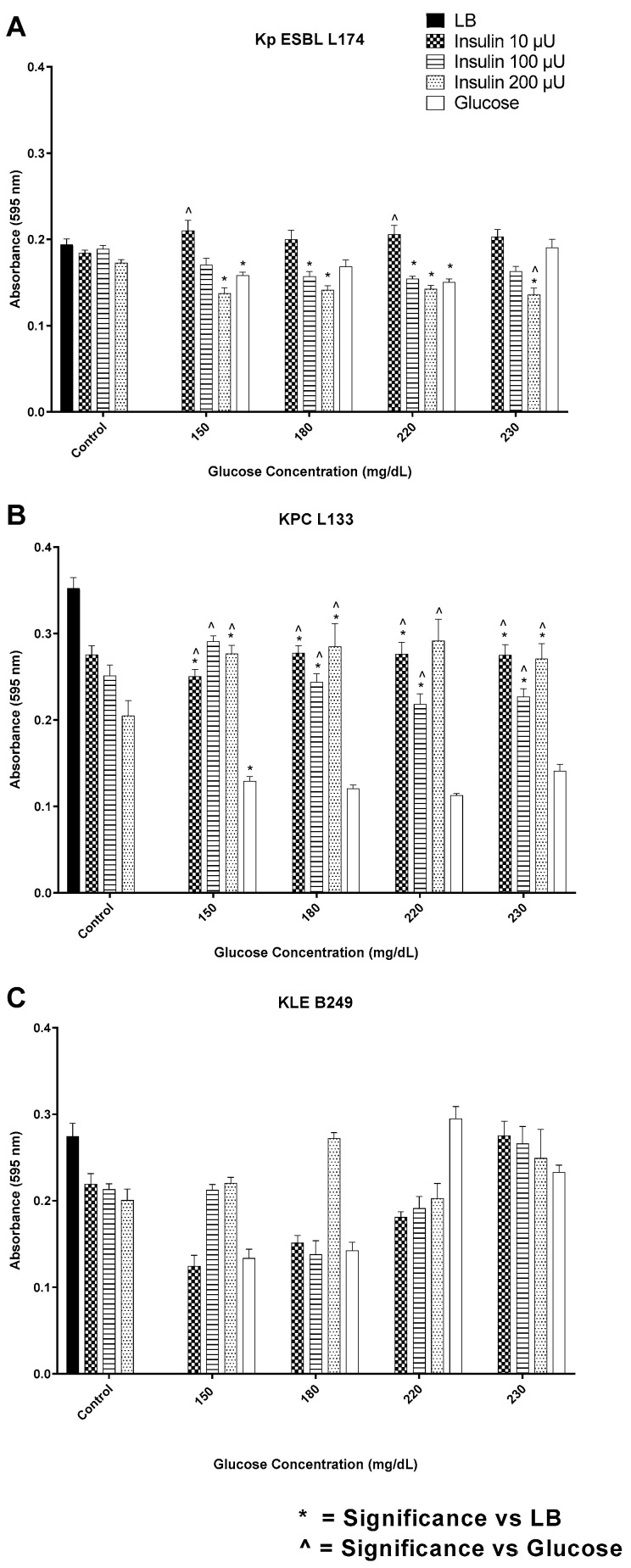
Biofilm formation by *K. pneumoniae* clinical isolates (**A**–**C**) in response to a range of insulin/glucose concentrations. * Significantly different (*p* ≤ 0.05) as compared to LB. ^˄^ Significantly different (*p* ≤ 0.05) as compared to glucose. Positive controls for biofilm formation were growth in LB alone, each insulin concentration in LB alone, and glucose in LB alone.

The response of the three multidrug-resistant clinical isolates of *K. pneumoniae* screened for their biofilm production response to insulin/glucose or glucose alone is shown in Figure 9A–C.

**Figure 10:**
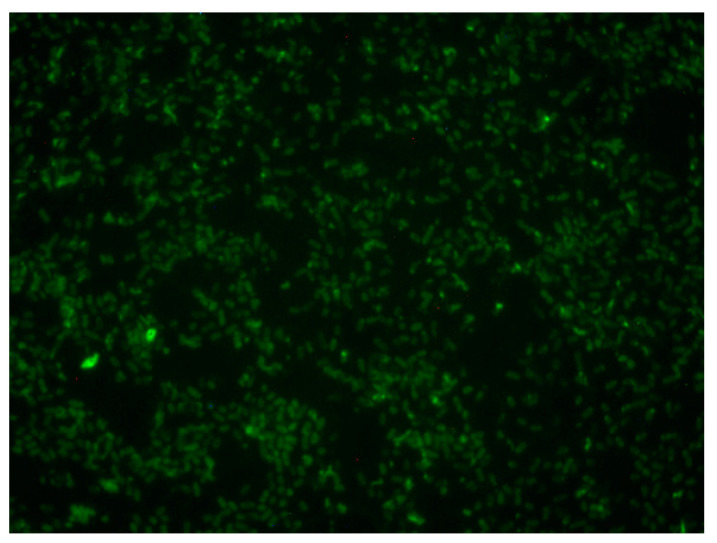
Binding of FITC-insulin to aerobically grown *K. pneumoniae* carbapenemase-producing (KPC) L133 cells after 18 h of aerobic growth.

For all three isolates, insulin affected biofilm expression in an insulin/glucose-specific ratio. For *K. pneumoniae* ESBL L174, regardless of glucose concentration below 230 mg/dL, insulin at 10 µU/mL enhanced biofilm to a similar extent, while 200 µU/mL insulin at 230 mg/dL glucose suppressed biofilm expression (Figure 9A). In contrast, for isolate KPC L133, the insulin/glucose ratio over the range of insulin concentrations enhanced biofilm production as compared to glucose alone control (Figure 9B). This concentration-specific insulin regulation of biofilm level was most prominent with isolate *K. pneumoniae* B249 (Figure 9C). At 180 mg/dL glucose, the presence of 200 µU/mL insulin induced a biofilm level similar to that of the maximal levels measured for this isolate. However, the insulin-induced variability was absent at 230 mg/dL glucose, where the biofilm levels measured for three insulin concentrations were similar to that of glucose alone. Like the other Gram-negative and Gram-positive bacteria tested, *K. pneumoniae* L133 was able to intrinsically bind FITC-insulin in a heterogenous pattern (Figure 10).

**Figure 11:**
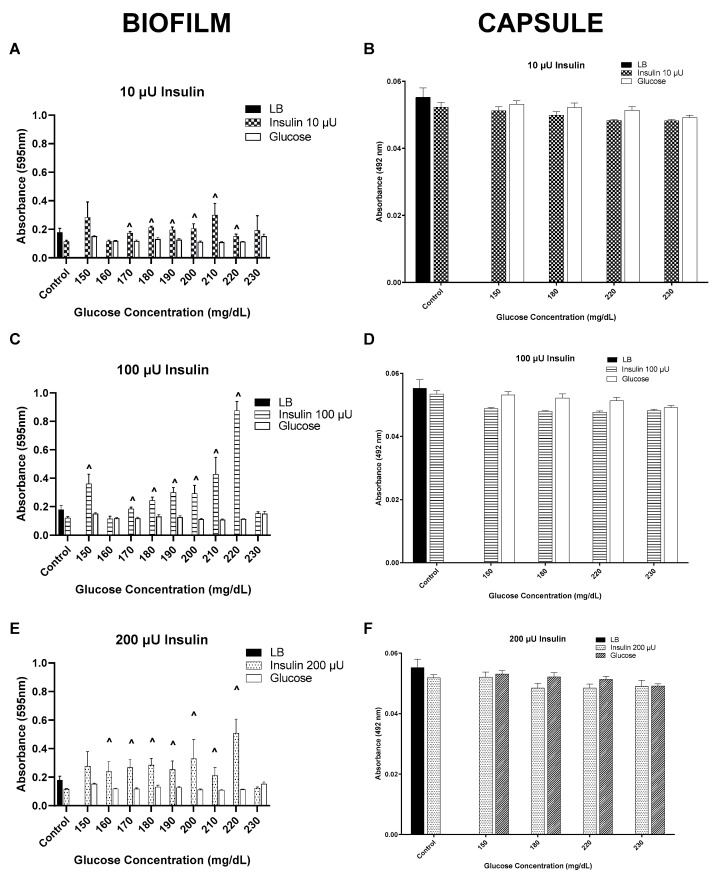
Biofilm (crystal violet staining; (**A**,**C**,**E**)) and capsule (Alcian blue staining; (**B**,**D**,**F**)) formation by *K. pneumoniae* ATCC 27736 in response to a range of insulin/glucose concentrations. ^˄^ Significantly different (*p* ≤ 0.05) as compared to glucose. Positive controls for biofilm formation were growth in LB alone, each insulin concentration in LB alone, and glucose in LB alone.

Since sepsis is associated with capsule formation, whether there is a correlation between biofilm formation and capsule production with respect to response to the insulin/glucose ratio for *K. pneumoniae* was examined using a highly stable encapsulated strain of *K. pneumoniae* ATCC 27736 (Figure 11A–F) [9,22]. As with the clinical isolates, biofilm formation by this isolate was affected by the presence of insulin at all concentrations tested (Figure 11A,C,E). In contrast, the production of the acidic polysaccharide capsule was unaffected by either insulin or glucose concentrations (Figure 11B,D,F).

## 4. Discussion

The hypermetabolic stress response of hyperglycemia has been shown to be closely associated with injury, trauma events, and/or acute hospital presentation [23,24,25,26]. Hyperglycemia during these hypermetabolic stress states not only poses a danger for individuals diagnosed with diabetes but represents a high critical risk for those with no known previous risk for diabetes which can account for 12% of adult patients admitted [23,24,25,26]. Beyond the known and undiagnosed diabetic individuals are the remaining trauma patients who respond to the traumatic assault with intrinsic transient hyperglycemia.

The mechanism underlying hyperglycemia is complex and includes metabolic and hormonal changes associated with increased circulating counterregulatory hormones and proinflammatory cytokines along with exogenous interventions, including the use of vasopressors, corticosteroids, and non-oral (enteral and parenteral) administration of nourishment [26]. Regardless of the underlying cause, the occurrence of hyperglycemia in acute hospitalized patients has with it increased morbidity and mortality, including that of sepsis [27]. This risk of complications and mortality is correlated with the extent of hyperglycemia. Interestingly, the level of trauma-associated hyperglycemia is typically higher in patients without known diabetes [23,28]. The increased risk from hyperglycemia including that of sepsis and multiorgan failure is empirically controlled with intensive insulin therapy [7,29,30,31]. This is the first study to systematically examine how the interaction of insulin with glucose over the physiologic range affects the expression of a virulence factor known to play a role in sepsis [32,33].

Insulin is a phylogenetically ancient protein not only synthesized across the taxonomic kingdoms but regardless of which source, exhibits cross-kingdom activity [13,14,15,16,34,35,36,37,38,39]. Although *E. coli* has been demonstrated to synthesize its own insulin, as well as respond to human insulin, it has only been recently that its role as a behavioral modifier in *E. coli* phenotypic expression regulation has been described [10,17]. Insulin can function as an interkingdom quorum signaling compound that affects the expression of biofilm formation in *E. coli* in a manner that is nutrient and environmentally sensitive [17,40,41,42,43]. Furthermore, in *E. coli*, human recombinant insulin (rh-insulin), like other quorum chemical signaling compounds, has been shown to play an important role in the regulation of *E. coli* chemotactic responses and growth rates, in addition to biofilm formation [10,12,41,42,43]. Of interest is that the ability of rh-insulin to function as an interkingdom quorum signal compound is not limited to *E. coli* but has also been demonstrated in *S. aureus* [11,44]. However, the response of pathogens associated with sepsis, particularly multidrug-resistant organisms wherein the presence of biofilm would decrease clinical treatment success in trauma, has not been examined.

This is the first report of the widespread ability of representative strains of blood-borne pathogens associated with sepsis to intrinsically bind insulin, regardless of cell wall architecture, i.e., Gram-positive or Gram-negative. In addition, with respect to the Gram-negative bacteria, there were cells that exhibited distinct patterns of binding: polar, punctate, and even. Of note is the preliminary finding that significant effects of insulin/glucose on biofilm formation do not appear to be a requirement for the binding of FITC-insulin to cells as shown by the photomicrograph of *A. baumannii* 185. Although we have not observed a lack of insulin binding regardless of species or cell wall architecture (Gram-positive or Gram-negative), this apparent receptor binding by insulin requires further study with the putative receptor characterized. A consideration pertinent to the insulin receptor is that the distribution of receptors could be linked to specific types of actions, e.g., motility–chemotaxis representing a more planktonic behavior, vis-à-vis sessile, or biofilm-associated behaviors. Especially notable are the reports of polar clustering of chemotactic receptors [45,46,47,48]. Interestingly, insulin receptors can exhibit a similar polar pattern of clustered receptors as reported for *E. coli* chemotactic receptors. This observation is intriguing since insulin has not been reported as a chemotactic agent for Gram-negative bacteria. It also indicates that exploitation of this receptor-signaling expression may provide an avenue for prevention of biofilm formation, or the potential conversion of biofilm sessile populations to planktonic populations that would be conceivably easier to eliminate from the host.

With regards to biofilm formation, the response of the organisms to insulin/glucose across physiologic insulin/glucose relevant concentrations was highly variable, dependent on the isolate, insulin level, and glucose concentration. Overall, the general pattern for all microbes was that glucose at 200 mg/dL, regardless of insulin level, induced the highest amount of biofilm. This correlates with the empirical findings that treatment of trauma patients to maintain glucose levels below 200 mg/dL decreases the risk for sepsis. The biofilm formation ranged from insulin (10 µU/mL) suppression of biofilm as compared to glucose alone at 180 mg/dL (MRSA T51995) to enhanced biofilm production at the same insulin concentration at 230 mg/dL glucose. In contrast, for VRE, insulin inhibited biofilm formation in a concentration-specific manner, maintaining constant biofilm levels regardless of the glucose concentration present. Suppression of biofilm was also notable for *A. baumannii* L187 at all insulin concentrations compared to glucose alone. However, of greatest concern were the effects of insulin on *Klebsiella* isolates with insulin causing enhanced production of biofilm as compared to glucose alone. The potential use of trauma protocol treatment enhances the presence of biofilm formation in a bacteremic individual could have an adverse outcome on antibiotic treatment due to the protective effects of biofilms [49].

The mechanism by which insulin/glucose modulates biofilm formation is not known. It is interesting that the differential staining of an encapsulated organism, *K. pneumoniae* provides some indication that the biofilm components modulated by insulin/glucose are not likely the acidic polysaccharide (Alcian blue stainable) component, but may be more related to the crystal violet (gentian violet) stainable biofilm components, e.g., protein, lipids and nucleic acids [20,50]. Taken together, these findings lay a foundation that indicates that although empirical administration of insulin in hyperglycemic acute trauma patients can be somewhat effective, there is also the potential that without knowledge of how the insulin/glucose can affect patients who may transition into sepsis, adequate antibiotic treatment could be hampered, particularly if the isolate was both multidrug-resistant as well as phenotypically induced to express higher levels of biofilm. This scenario could unfortunately also function to synergistically select for increased drug resistance [51]. Our initial study points out the need for the development of hormone biofilmgrams analogous to the antibiograms currently in clinical usage for predictive use in a septic event. In addition, it points out the need to develop real-time insulin measurement for use with constant glucose monitoring since together with the institution of clinical microbial biofilm screening, there is the potential to decrease patient morbidity and mortality, as well as overall hospital costs in trauma patients.

## 5. Conclusions

In both critically ill and noncritically ill patients, extremes in blood glucose levels tend to lead to poor outcomes. However, glycemic targets obtained through the administration of insulin can be difficult to maintain since critically ill individuals exhibit metabolic instability with an inclination to shift insulin requirements. A risk in this patient population is the development of bacteremia with subsequent initiation of infection site colonization with biofilm formation. Although previous studies have shown that for a select few of the bacteria examined, their response to insulin and or glucose with respect to biofilm formation was insulin and/or glucose-concentration specific, we have demonstrated in this foundational study that not only do organisms representing both Gram-positive and Gram-negative cell wall architectures exhibit an intrinsic ability to bind insulin, but that their response to insulin/glucose ratios is also species and strain specific. Hyperglycemia is associated with infectious morbidity in hospitalized and trauma patients. Since this metabolic stress response is an independent predictor of patient outcome, as is sepsis with both associated with increased mortality in the trauma population, there is the potential that modulation of the insulin/glucose ratio, while it may contribute to poorer patient outcome, could also be modified in a real-time clinical setting to suppress the formation of biofilms with the potential of affecting a positive patient outcome. Further studies expanding these findings as well as the development of real-time in situ insulin measurement probes are in progress.

## Data Availability

Data are contained within the article.
